# Is there a place for undergraduate and graduate students in the systematic review process?

**DOI:** 10.5195/jmla.2018.387

**Published:** 2018-04-01

**Authors:** Christina L. Wissinger

**Affiliations:** Assistant Librarian and Health Sciences Liaison, Pennsylvania State University Libraries, University Park, PA

## Abstract

Systematic reviews are a well-established and well-honed research methodology in the medical and health sciences fields. As the popularity of systematic reviews has increased, disciplines outside the sciences have started publishing them. This increase in familiarity has begun to trickle down from practitioners and faculty to graduate students and recently undergraduates. The amount of work and rigor that goes into producing a quality systematic review may make these types of research projects seem unattainable for undergraduate or graduate students, but is this an accurate assumption? This commentary discusses whether there is a place for undergraduate and graduate students in the systematic review process. It explains the possible benefits of having undergraduate and graduate students engage in systematic reviews and concludes with ideas for creating basic education or training opportunities for researchers and students who are new to the systematic review process.

In the last year, I have noticed a substantial increase in the number of graduate students who have been sent to me because their faculty advisor told them they need to do a systematic review. This was more of an oddity than a concern until undergraduate students started coming to me because they had received the same advice. My first reaction was “What are they thinking?!” While that thought still flows through my mind, I am starting to think about this new occurrence strategically. Is there a place for undergraduate and graduate students in the systematic review process? Can we use this as an opportunity to educate and perhaps increase the quality of published systematic review [1]? As we know, not all systematic reviews are created equal [2].

What follows is my best attempt to outline the pros and cons of having students engaged in the systematic review process from my perspective as a health sciences librarian in a general academic library and to share my ideas for using this confusion as an opportunity to enhance informal systematic review education.

Let me start with the limitations: Because the typical academic semester lasts around fifteen weeks, it is almost impossible for an undergraduate student to complete a systematic review as part of a course assignment. For undergraduate students who complete an honors thesis or a capstone project, a systematic review might be a more achievable goal, but ending up with something that is completed well enough to be considered for publication is unlikely.

The most difficult barrier for undergraduates to overcome is that a high-quality systematic review needs at least two people to review the retrieved articles [3]. If the systematic review was an individually graded assignment rather than a group project, it would be awkward for students to find someone else to help them complete this part of the process. Also, students need to become proficient with citation management software to organize their articles and remove duplicate records and must adequately document the process to be able to fill out the PRISMA flow chart [4].

Ultimately, it may not be realistic to expect undergraduate students to take on a project this time-consuming and have them keep up with the pace of their regular course work. Although graduate students can overcome most of these obstacles, we need to ask ourselves if it is realistic for them to complete a systematic review as part of their comprehensive exams or dissertations. Typically, theses and dissertations include an element of original research or experimentation. If the thesis or dissertation itself is not a systematic review, asking graduate students to complete a systematic review instead of a literature review, in addition to completing original research or experiments, might be overkill. If the full thesis or dissertation is a systematic review or meta-analysis and no additional work is required, then this expectation is more realistic [5].

Now for the benefits: Even if undergraduate students cannot complete a full systematic review, perhaps having them go through the search construction and retrieval phase will enhance their skills related to selecting appropriate databases, mining for keywords and controlled vocabulary terms, applying relevant limits, and exporting citations. These are all valuable skills that would help make students critical consumers of published systematic reviews, as they would gain firsthand experience with a key part of the process. Over time, this experience could lead to an increase in the quality of published systematic reviews because these students would be able to identify a systematic review that fell short of the standards [1, 2].

To consider another perspective, many undergraduate students in the health sciences go on to apply for medical school or graduate programs, and having experience with performing systematic reviews may give them a competitive advantage during the interview process. Being able to competently discuss a sophisticated research process shows their potential in taking on new challenges and their initiative in acquiring advanced skills [6]. Also, this opens up a new pathway for librarians to connect with undergraduates outside of the traditional one-shot library session and build long-term relationships.

As systematic reviews have gained in popularity, fields in the social sciences have also begun publishing resources for students and faculty who are interested in the process [7]. As a health sciences librarian in a general academic library, I have consulted with students in education who were working on a systematic review and whose adviser had no hands-on experience conducting this type of research. Thus, librarians could use this expansion into other disciplines as an opportunity to create campus-wide educational programing that is relevant to areas beyond the health sciences.

The first step might be an all-inclusive lecture, during which researchers in fields beyond health and medicine can learn from systematic review experts. This outreach opportunity could be paired with new faculty orientation, graduate student orientation, undergraduate honors student orientation, undergraduate or graduate research days, or institutional review board training. This type of outreach programing would allow participants to have a better understanding of what they are being asked to do or what they are asking their students to undertake.

We could take this further by creating a mock peer-review process where high- and low-quality published systematic reviews are evaluated, with experts pointing out the differences. This experience could be included in graduate and undergraduate courses or programs in which students are expected to produce an in-depth research paper like a capstone, thesis, or dissertation. Additionally, we could partner with on-campus research centers that provide additional training opportunities to faculty, staff, and students to conduct small hands-on systematic review workshops. Participants could first evaluate systematic reviews, and then experts could facilitate group discussions and share their critiques.

I still have no idea why faculty are telling undergraduate students to conduct systematic reviews or why graduate students are expected to do a systematic review as part of their comprehensive exams or dissertations. Perhaps the “why” is not important; maybe we need to accept this as an opportunity and use it to our advantage. At the end of the day, students are being sent to the library by their faculty, and we are given another venue to work with students and enhance their research skills.

## References

[1] Koffel JB, Rethlefsen ML (2016). Reproducibility of search strategies is poor in systematic reviews published in high-impact pediatrics, cardiology and surgery journals: a cross-sectional study. PLOS ONE.

[2] Schardt C Are all systemic reviews created equal? [Internet].

[3] Edwards P, Clarke M, DiGuiseppi C, Pralap S, Roberts I, Wentz R (2002). Identification of randomized controlled trials in systematic reviews: accuracy and reliability of screening records. Statist Med.

[4] PRISMA Flow diagram [Internet].

[5] Boland A, Cherry MG, Dickson R (2014). Doing a systematic review: a student’s guide.

[6] Albanese MA, Snow MH, Skochelak SE, Hugget KN, Farrell PM (2001). Assessing personal qualities in medical school admissions. Acad Med.

[7] Petticrew M, Roberts HS (2006). Systematic reviews in the social sciences: a practical guide.

